# Chemotherapeutic Drug Delivery Nanoplatform Development: From Physicochemical to Preclinical Evaluation

**DOI:** 10.3390/ijms252111520

**Published:** 2024-10-26

**Authors:** Orestis Kontogiannis, Dimitrios Selianitis, Konstantinos Palikaras, Natassa Pippa, Stergios Pispas, Efstathios Efstathopoulos, Maria Gazouli

**Affiliations:** 1 Department of Basic Medical Science, Laboratory of Biology, School of Medicine, National and Kapodistrian University of Athens, 11527 Athens, Greece; ore-ko@windowslive.com; 2 Theoretical and Physical Chemistry Institute, National Hellenic Research Foundation, 48 Vassileos Constantinou Avenue, 11635 Athens, Greece; dimitrissel404@gmail.com (D.S.); pispas@eie.gr (S.P.); 3 Department of Physiology, School of Medicine, National and Kapodistrian University of Athens, 11527 Athens, Greece; palikaraskon@gmail.com; 4 Department of Pharmaceutical Technology, Faculty of Pharmacy, Panepistimioupolis Zographou, National and Kapodistrian University of Athens, 15771 Athens, Greece; natpippa@pharm.uoa.gr; 5 2nd Department of Radiology, Medical School, Attikon University Hospital, National and Kapodistrian University of Athens, 11527 Athens, Greece; stathise@med.uoa.gr

**Keywords:** Pluronic 188, MTS assay, thin-film hydration, 3D cell culture, real-time PCR, *C. elegans*

## Abstract

Through this study, the synergistic behavior of small-molecular-weight, amphiphilic surfactant molecules and the triblock copolymer Pluronic 188 was extensively evaluated based on their ability to formulate nanocarriers with novel properties for the delivery of class II and IV (biopharmaceutical classification system) chemotherapeutic compounds. The combination of four different surfactants at multiple weight ratios and twelve initially formulated nanosystems resulted in four hybrid delivery platforms, which were further studied in terms of multiple physicochemical characteristics, as well as their stability in protein-rich media (fetal bovine serum/phosphate-buffer saline). Finally, we obtained a single final nanoformulation that exhibited a high loading capacity (%EE ≥ 75%) and a sustained drug release profile under physiological conditions (model drug methotrexate), without altering the original physicochemical characteristics of the carrier. With a mean hydrodynamic radius (Rh) of less than 70 nm, a polydispersity index of 0.219, and no protein complexation, the system is a suitable candidate for in vivo, intravenous, and/or intramuscular administration. The cytotoxicity and genotoxicity of both loaded and unloaded carriers were evaluated through the examination of the upregulation or downregulation of apoptosis-related pathways. Multiple conventional 2D and 3D spheroidal conformations were used for these assessments, including HEK293, HCT-116, and MCF-7 cell lines, the results of which stressed the safety and biocompatibility of the empty nanocarrier. Additionally, experiments on *Caenorhabditis elegans* were conducted to evaluate the system’s in vivo toxicity, focusing on developmental stages, egg-laying behavior, and locomotion. Nanosystems studied in terms of chemotherapeutic encapsulation have mostly focused on the physiochemical aspect of the development of such novel delivery platforms, with only few exceptions proceeding step-by-step from cellular 2D to 3D to in vivo experimentation. The present study offers a holistic view of the behavior of such a novel system, advancing our understanding of the capabilities of polymeric/surfactant-based nanodelivery platforms.

## 1. Introduction

The formulation of advanced, novel, and biocompatible nanosystems to be used towards cancer treatment and/or diagnosis is a research field that holds great promise, especially with regard to the development of therapeutic approaches against solid tumor proliferation (≥90 percent of human adult cancers are solid tumors) that combine more conventional treatment plans with more advanced methods [1,2,3]. Block copolymers are able to produce highly monodisperse nanostructures, along with the ability to encapsulate both hydrophilic and lipophilic active pharmaceutical ingredients (APIs) with a relatively high loading capacity. Often resulting in amphiphilic structures with biomimetic internal compartmentalization, a process initiated via the spontaneous self-assembly of such advanced material when dispersed in aqueous media, they can create complex architectures with multiple compartments [4,5,6,7]. Members of the Pluronics family (also called poloxamers or superonics) consist of non-ionic amphiphilic triblock copolymers with different PEO/PPO chain ratios, affecting each copolymer’s ultimate behavior in terms of microphase separation, aqueous solubility, bioavailability, and loading capacity.

Surfactants are molecules that hold great promise in the pharmaceutical industry due to their ability to increase the solubility—and, as a result, the bioavailability—of poorly dissolved drug components. At the same time, they effectively minimize the surface tension between a delivery platform and the cellular membranes, enhancing cellular adhesion and drug permeability [8,9]. The utilization of non-ionic monomeric counterparts in the formulation of the hybrid nanosystem prolongs circulation time by minimizing the system’s non-specific interactions with components of the innate circulation such as albumin. In turn, this characteristic diminishes immune system recognition, which can result in rapid renal excretion [10].

Methotrexate (MTX) is considered to be one of the first approved anti-metabolite, cytostatic drugs and has been widely used in recent decades with measurable success towards cancer treatment (breast cancer, leukemia, head and neck cancer, osteosarcoma, etc.). As a compound, MTX has the ability to deactivate the metabolism of deceased tumorous cells through the mechanism of apoptosis by ceasing intracellular folate metabolism. As a result, MTX disrupts the synthesis of thymine and purines also leading to secondary genotoxic effects by interrupting both DNA and RNA synthesis. Higher doses of MTX are considered the only alternatives to a standard CHOP regiment (cyclophosphamide, doxorubicin, vincristine and prednisone), especially for more aggressive cancerous subtypes that exhibit poor clinical improvement, attributed partially to the compound’s short plasma half-life, and the high antidrug resistance that such subtypes present [11]. Unfortunately, large doses of MTX, distributed throughout an organism lacking tissue selectivity, exhibit a high side-effect profile, including myelosuppression, hepatotoxicity, pneumonitis, emphysema, leukopenia, and nephrotoxicity, while unwanted drug accumulation in excretory organs is responsible for additional toxicity-related problems. Blood serum levels regarding free methotrexate administration are generally not detectable after 18 h (with an approximately 6 h half-life), while for low-dose administration, the half-life ranges from 3 to 10 h. This in turn affects the therapeutic protocol that a patient has to follow, shortening the interval between two subsequent treatments. This can have a negative effect on patient compliance and phycological well-being (more often hospitalizations). Delivery platforms that exhibit high homogeneity and sizes in the nanometer range (i.e., ≤200 nm in terms of hydrodynamic diameter) are able to utilize the enhanced retain and permeability (EPR) effect, taking advantage of the rapid cancer cellular proliferation that results in the formation of bigger gaps between the endothelial cell membranes in comparison to healthy cells. This phenomenon allows nanosystems, given enough circulation time, to reach the tissue of interest, resulting in a type of selective permeation [12].

The overall aim of this study was the successful incorporation of MTX in novel, hybrid and biocompatible polymeric nanosystems, with improved drug loading, distribution, and drug release characteristics, along with a lower side-effect profile when compared with the administration of the free drug.

With respect to intravenous administration, it is crucial for the selected nanosystem to maintain its physicochemical characteristics, drug release profile, and immune response, unaffected by protein corona formation (both the appearance of a soft and hard corona, with the latter exhibiting stronger particle–protein interactions); therefore, its stability in simulated physiological conditions needs to be determined. Measurements using dynamic light scattering (DLS) were utilized to determine the protein–nanosystem interactions of the final formulation when dispersed in fetal bovine serum along with mixing the nanoformulation with 10% FBS/PBS solution after prolonged exposure times [13,14,15,16,17,18,19]. Herein, we report the final formulation of Pluronic 188–Tween 80–MTX (9:1:0.2), having been extensively studied in terms of its physicochemical characteristics and stability, encapsulation efficiency, and the release properties of the physically entrapped MTX in various conditions. Several characterization methods were utilized, such as DLS and UV–Vis spectroscopy, while in vitro cytotoxicity assays were performed in both monolayer cellular cultures and 3D spheroidal conformations. Lastly, in vivo toxicity experiments were conducted using wild-type *Caenorhabditis elegans* nematodes, effectively bridging the gap between in vitro experiments and more complex in vivo models. *C. elegans* is a simple multicellular animal model extensively used in chemical and genetic screens. This nematode offers several culture-related advantages, including low cost, a short lifespan of approximately 2–3 weeks, and a brief reproduction cycle. Additionally, it exhibits many molecular-level similarities with humans. Each nematode can produce up to 300 progenies through self-fertilization, and this number can increase to up to 1000 new worms when fertilization involves a male (XO). To the best of the authors’ knowledge, this is the first time that methotrexate has been encapsulated in a block copolymer/surfactant nanosystem such as this; thus far, most experimental protocols have focused on MTX encapsulation in the hydrophobic core of mixed-block copolymer micellar nanosystems, without the use of surfactants (mostly P127/PC, P127/P105 systems). Other studies examined nanoformulations of various surfactant mixtures (Tween 80 and Span 80) but without the use of block copolymers [11,17,18,19,20,21,22,23].

## 2. Results and Discussion

### 2.1. Physicochemical Characterization and Stability Assessment of the Formulated Nanosystems After Filtration

The physicochemical investigation of the stability of the most promising nanosystems in physiological conditions was evaluated based on the size, size distribution, polydispersity index, and scattered intensity, as well as the microfluidity and microviscosity characteristics that were previously reported [16]. Through the self-assembly process that such amphiphilic systems present when exposed to aqueous media (minimization of the surface area of the hydrophobic parts of the polymeric chain “available” to interact with water), conformations of complex internal structures can be generated, providing the system with unique attributes (internal compartmentalization, loading capacity, increased toxicity, etc.). DLS was used in order to evaluate the nanoparticulate colloidal dispersion’s stability in FBS/PBS media after incubation for at least 1 h.

In Figure S1, the stability characteristics are presented for the following nanosystems: Pluronic 188–Tween 80 (90:10); Pluronic 188-Span 40 (90:10); Pluronic 188-Span 40 (50:50); and Pluronic 188-Span 60 (50:50). The existence of non-specific interactions between the proteins of the media and the hybrid nanosystem results in the formation of a protein corona that alters the original physicochemical characteristics of the system, such as the size and PDI, resulting in the formation of a new peak in the resulting graph. Since the final formulation needs to be inert in order to avoid immune system recognition and circulate for a prolonged period of time to reach the target tissue, the nanosystems that indicate the occurrence of non-specific interactions after FBS/PBS incubation are not optimum for human applications. Such complexation results in a final nanosystem with different architectural characteristics and three-dimensional conformation, raising a plethora of concerns in terms of its safety profile and possible inactivation of successful ‘payload’ delivery, providing the same clinical effect. Larger particles, occurring from such protein interactions, increase the potential for distal organ toxicity (changes in the excretory route) and the possible blockage of smaller capillaries (resulting after repeated administration in thrombosis via the organism’s potential inability to clear the carriers at the same rate as administered).

### 2.2. Encapsulation of MTX and Characterization of Final Formulated Nanosystems

**Figure 1:**
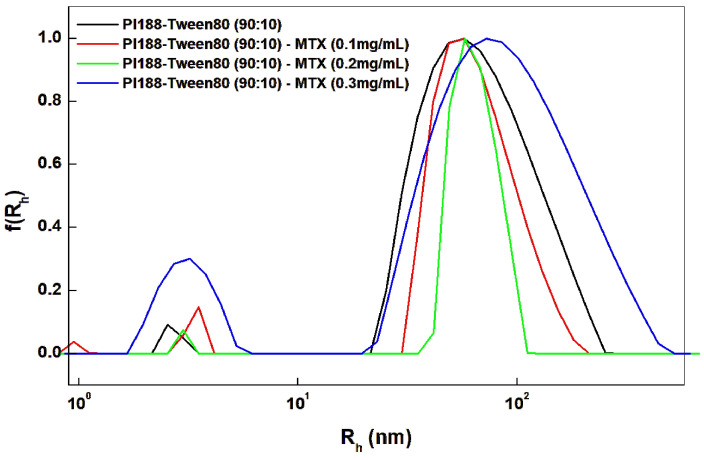
Comparative size distribution graphs of Pluronic 188–Tween 80 (90:10) nanosystems with MTX encapsulated from 0.1 mg/mL up to 0.3 mg/mL and the filtered unloaded system of Pluronic 188–Tween 80 (90:10).

Proceeding towards API encapsulation, the selected model drug, methotrexate (highly lipophilic compound expected to be physically entrapped in the hydrophobic core of the amphiphilic nanosystem), was entrapped in three distinct concentrations ranging from 0.1 to 0.3 mg per mL of aqueous nanoparticle dispersion. Thin-film hydration was used for the development of the nanosystem (Figure 1). According to the hydrophilic-to-lipophilic balance (HLB) of the triblock copolymer, Pluronic 188, and the small-molecular-weight surfactant molecule, Tween 80, the amount of the lipophilic compounds that can be loaded is affected and somewhat limited in comparison to using more lipophilic molecules [8,9]. The micellar stability must be considered when choosing the final formulation in terms of drug/block copolymer ratio, with an encapsulation ratio above 3:92 producing systems with higher precipitation rates, lower stability over time and an overall higher hydrodynamic diameter [11,20]. This phenomenon can be attributed to the increased hydrophobicity of the system’s core, which can no longer be supported by the hydrophilic outer periphery of the micelle.

As shown in Table 1, for up to 0.2 mg/mL of MTX incorporation in the final nanosystem, the hydrodynamic radius and polydispersity of the formulation are decreased. Until that point, drug accommodation in the core is taking place due to the large interactions of the lipophilic chains, and as such, the system is prone to producing more homogenous systems, suitable for intravenous and intramuscular administration. The formulated nanosystem is a result of mixing at different weight ratios the triblock copolymers, Pluronic 188 and Tween 80, both amphiphilic molecules but with relatively high HLB (with a stronger hydrophilic component). Up to a certain concentration of a hydrophobically encapsulated API, this favors the formulation in terms of avoidance of agglomeration; at the same time, it prevents the nanosystem from being able to encapsulate a higher amount of MTX. At a concentration higher than 4:96 MTX in a block copolymer, the obtained nanosystems exhibited unfavorable physical characteristics and reduced stability due to rapid precipitation [11]. Nanosystem development concerning 0.2 mg/mL MTX encapsulation was replicated thrice, validating the reproducibility of the results in terms of size, size distribution, and the polydispersity index (PDI) of the obtained colloidal dispersion (Figure S2).

**Figure 2:**
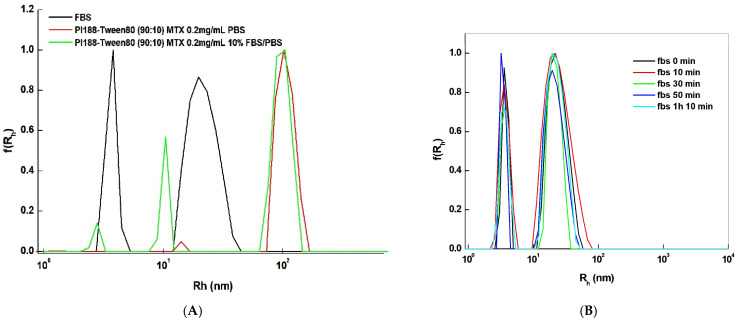
Size distribution graphs after incubation in physiological conditions. (**A**) The system’s stability assessment after 1 h incubation. (**B**) Incubation in fetal bovine serum where after 1 h the system was continuously monitored.

In terms of developing a final formulation intended for in vivo drug delivery applications, the system was extensively studied in physiological conditions. As exhibited in Figure 2, the system retains its physicochemical properties after incubation for a prolonged period (1 h at 4 °C, following an additional 1 h 10 min at a stable room temperature) in media with a high concentration of BSA.

Lastly, the colloidal stability of the final formulation over time was assessed (4 °C), exhibiting a retention of its original physicochemical properties (size, size distribution, polydispersity index) for a period of at least 8 days post formulation (Figure S3). After the 8th day, a slight increase in the Rh was observed, which might be attributed to an aggregation of the hydrophobic cores of the nanosystems. After lyophilization, the formulation could most likely remain stable under good storage conditions and retain a significant amount of methotrexate for a period of 6 months [19]. The system’s ability to obtain its original architectural characteristics in terms of its size and size distribution, along with the ability to avoid complexation with proteins of the innate blood circulation, offers a unique double advantage that allows for the development of an intravenous therapeutic modality with great promise.

### 2.3. Attenuated Total Reflectance–Fourier-Transform Infrared Spectroscopy (ATR-FTIR)

In order to successfully identify the presence of methotrexate encapsulated in the hydrophobic core of the nanosystems, FTIR spectroscopy was utilized (Figure S4), through which the characteristics of the chemical structure of MTX molecules were observed in the utilized spectral range [21]. It is important to note that the original peaks of free-form methotrexate are expected to be slightly shifted due to the presence of the block copolymer/surfactant molecules and the MTX being encapsulated inside the hydrophobic core of the whole nanosystem [20].

### 2.4. Encapsulation Efficiency of Pluronic 188–Tween 80 Hybrid Nanosystems by UV–Vis Analysis

The content of the encapsulated API was quantified by using UV–Vis spectroscopy at an absorbance of 303 nm [22]. Prior to detection, an appropriate dilution in acetonitrile was performed, with the following equation describing the calculation process:

Encapsulation efficiency (%) = (amount of drug encapsulated in hybrid nanosystems/amount of drug initially used) × 100

For the calculation, the standard curve for MTX was established by measuring six different concentrations diluted in acetonitrile, with each sample having twice the concentration of the previous sample, ranging from 0.025 mg/mL up to 0.8 mg/mL. Table S1 summarizes the results of those experiments, which are in accordance with the measurements from DLS (Figure 1), indicating that a quantity above 0.2 mg/mL cannot be encapsulated by the nanosystem, without critically compromising the original physicochemical properties of the unloaded Pluronic 188–Tween 80 (90:10) filtered nanoformulation. The results were replicated thrice in order to further assess the reproducibility of the final formulation (Figure S5) with %EE remaining above 75% in all instances.

### 2.5. Release Studies

The release profile of the encapsulated API inside the hydrophobic core of the hybrid Pluronic 188–Tween 80 nanosystems was evaluated in phosphate-buffer saline (PBS, pH = 7.4) and in 1:9 FBS/PBS ratio (pH = 7.0). In this way, both normal physiological conditions (37 °C), as well as the physiologically average conditions, which typically occur in a cancerous microenvironment (40 °C), were simulated. At multiple time intervals (including 0 min, 15 min, 30 min, 45 min, 1 h, 1 h 30 min, 2 h, 3 h, 4 h, 5 h, 6 h, and 6 h 15 min), 3 mL aliquots were withdrawn and re-introduced in the medium at the end of the analysis in order to avoid affecting the overall concentration of the API each time.

**Figure 3:**
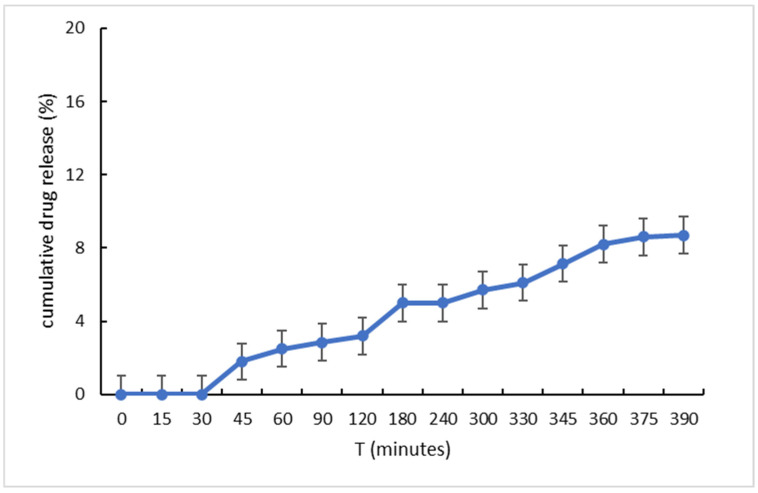
In vitro drug release study of MTX (303 nm) in PBS media at 37 °C.

The maximum concentration of MTX in the release medium was 2.0 μg/mL, while the average solubility in PBS (pH = 7.4) was about 40 μg/mL, creating optimal sink conditions [19]. About 0.015 mg/mL was estimated to have been released by 6 h (equal to 9% of the total physically entrapped MTX inside the dialysis bag; Figure 3 and Figure S6), indicating a sustained release rate from the hybrid nanosystems. This might suggest that the Pluronic 188–Tween 80–MTX nanosystem could be a chemotherapeutic nano-drug delivery platform that enables the system to deliver most of the encapsulated API passively in the tumor site, minimizing the exposure of healthy tissues to class II and IV highly toxic drug compounds.

### 2.6. MTS Cytotoxicity Assay

**Figure 4:**
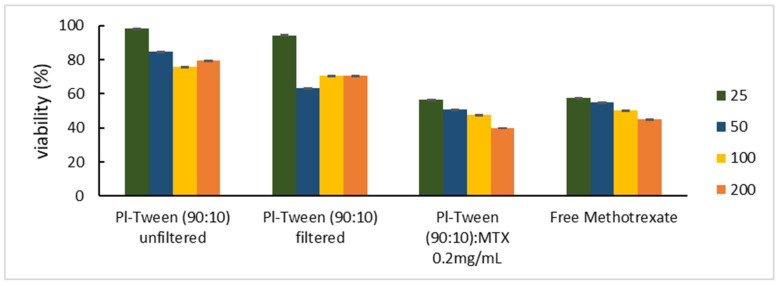
HEK293 adhesive cell line viability vs. different concentrations of the Pluronic 188–Tween 80 nanosystem, along with free methotrexate at equal concentrations as encapsulated in nano-dispersions. Each graph accounts for the dispersion of the viability percentage via the standard deviation. Results after a 24 h incubation.

With respect to the viability assessment conducted for the same unloaded nanosystems (unfiltered) that were presented in our previous study [16], comparative plot diagrams were formulated in order to assess the in vitro toxicity of the same nanosystem, both filtered through a 0.22 μm hydrophilic filter membrane and loaded with 0.2 mg/mL MTX. In Figure 4, the viability of the HEK293 adhesive cell line is shown (chosen as a normal human tissue cell model), with respect to the equal concentration of free MTX that the cells were exposed to. HEK293 cells represent a usual in vitro model in nanotoxicity evaluation, since they tend to have a sensitive response to nanoparticle exposure, are easy to cultivate, and have a widely validated experimental protocol accepted by the scientific community, partly due to their ease of transfection.

**Figure 5:**
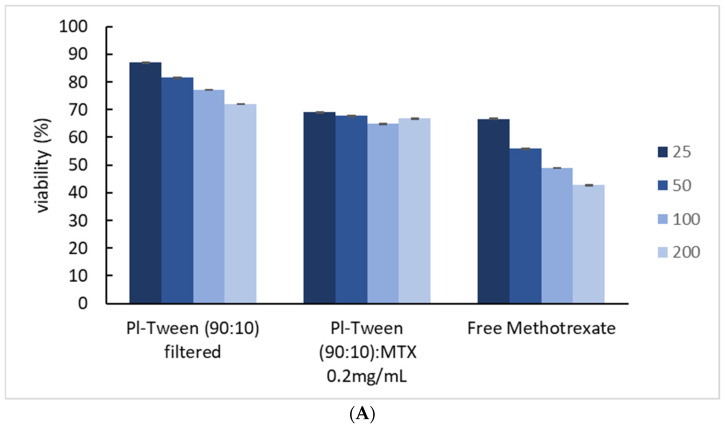

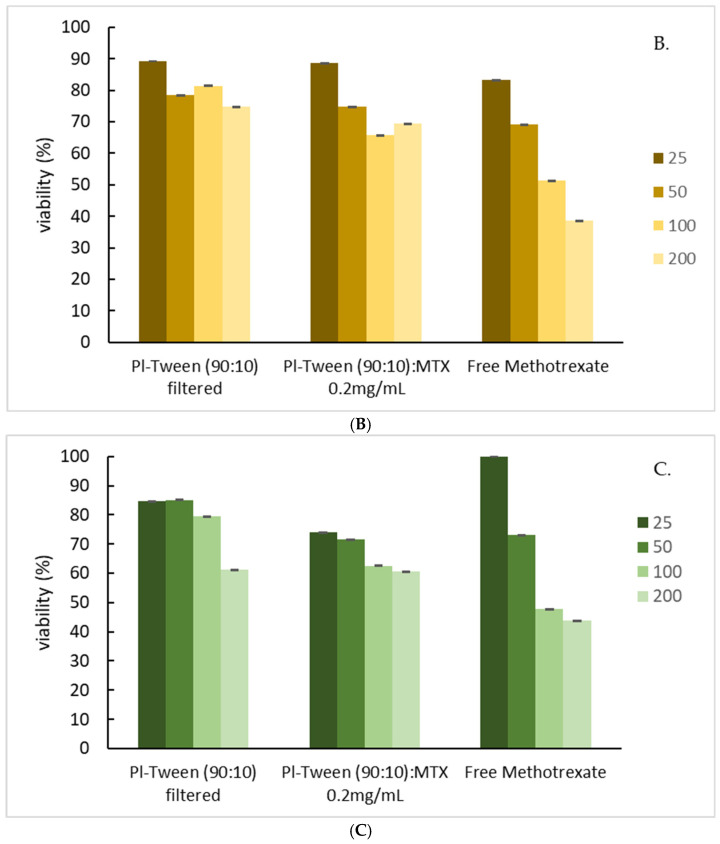
Cell viability vs. different concentrations of the Pluronic 188–Tween 80 nanosystem, along with free methotrexate at equal concentrations as encapsulated in nano-dispersions: (**A**) HCT-116 adherent human colon cancer cell line, (**B**) MCF-7 adherent human breast adenocarcinoma cell line, and (**C**) SK-BR3 estrogen-independent adherent human breast cancer cell line.

In all experiments, the maximum amount of MTX present in each cell culture was 4 μg/mL, with a maximum concentration of Pluronic 188–Tween 80 of 200 μg/mL. As exhibited in Figure 5, the unloaded nanosystem showed the least cytotoxicity (note that both amphiphilic components are biocompatible), while a dose-dependent toxicity was observed in all systems [23]. These values are expected to be lower than those from the 3D spheroidal cultures, since cells organized in monolayers (2D) are shown to be more susceptible to most chemotherapeutics [24,25,26,27].

It is worth paying attention to the results in Table 1, and specifically to the Rh (nm) values of the unloaded carriers, correlating this physical attribute with the ease of endocytosis and how it can correlate with the slightly reduced viability values. It is well established that nanoparticle cytotoxicity is inversely proportional to the size of the nanosystem. According to the classification of all non-ionic surfactants, at a concentration of even 1.0 mg/L, some reach their LC_50_ values (effectively killing 50 percent of a sample population). Translating the above concentration to the lab-scale values that we applied (96-well plate with 3.2 mL nominal volume in each well), and given the fact that each nanosystem is the result of more than one component (with the possibility of a slight increase in each counterpart’s inherent toxicity), we can conclude that even though they are biocompatible molecules, they are not without toxicity effects.

**Figure 6:**
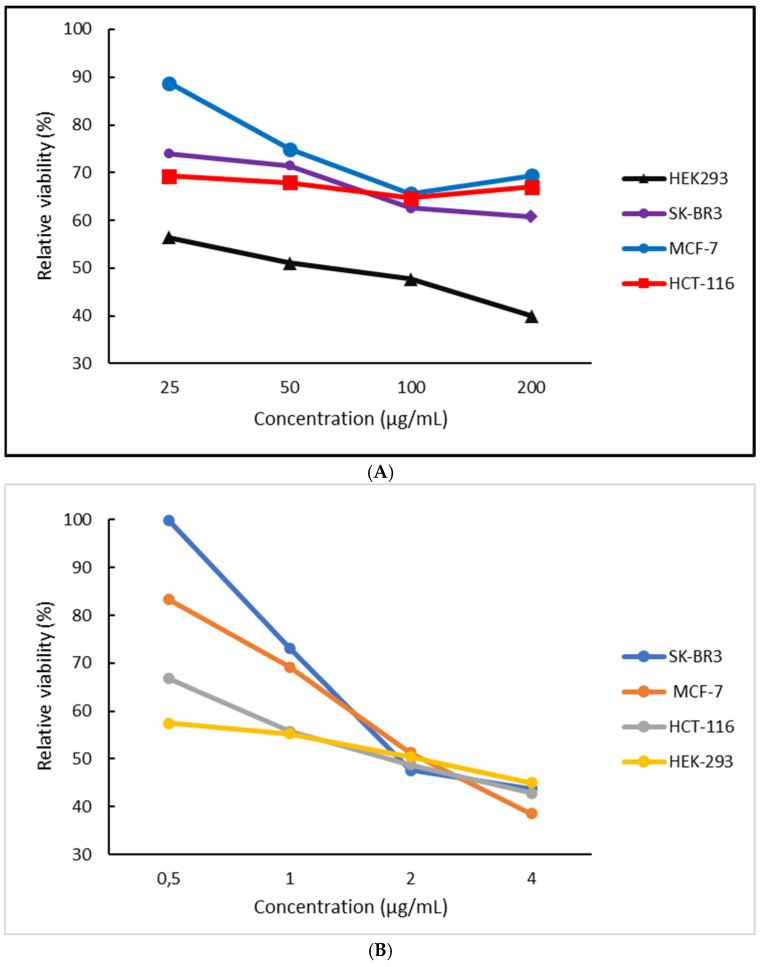
Relative viability (%) vs. the concentration (μg/mL) of (**A**) Pluronic 188–Tween 80–MTX (9:1:0.2)-loaded micelles in multiple cell line 2D cultures and (**B**) equal concentration of free MTX.

**Figure 7:**
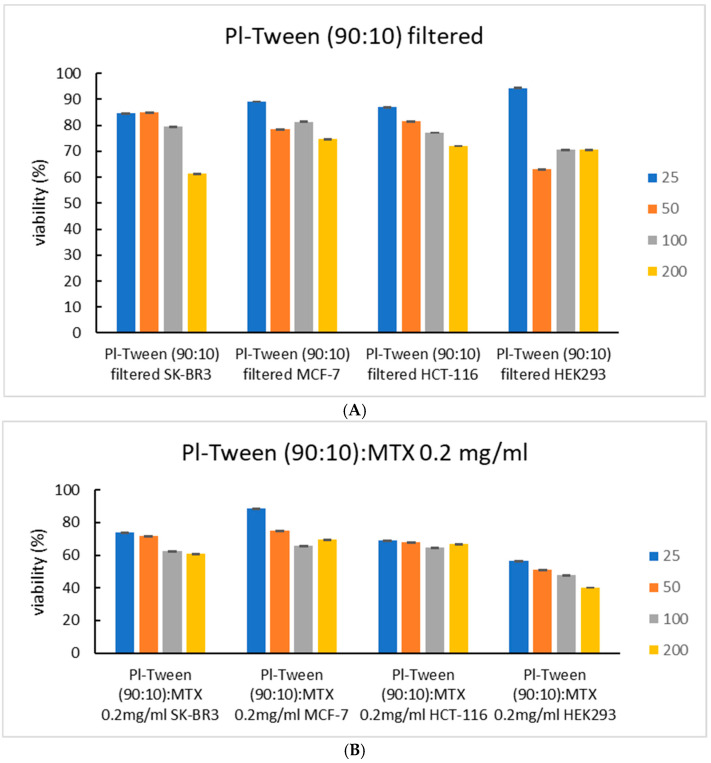

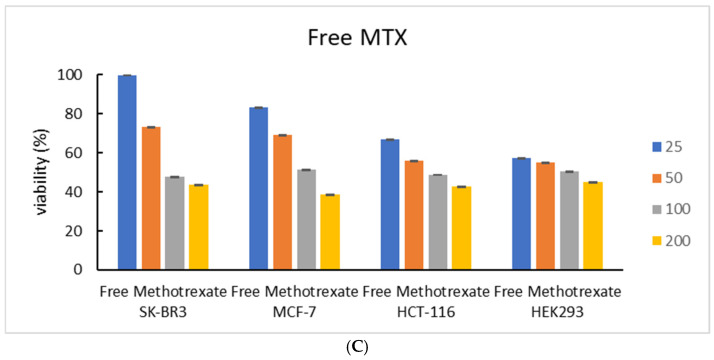
Cell viability expressed for the same nanosystem in relation to cell type incubation. Results after 24 h exposure, with concentrations ranging from 25 μg/mL to 200 μg/mL: (**A**) Pluronic–Tween 80 (90:10) filtered, unloaded nanosystems; (**B**) Pluronic–Tween 80 (90:10) nanosystems loaded with 0.2 mg/mL MTX; and (**C**) incubation with free MTX.

The type of cellular death that is depicted from MTS cytotoxicity experiments (Figure 6) cannot be determined without further experimentation since the assay detects mitochondrial metabolic activity and not signs of necrosis [11,21]. Figure 7 serves as an immediate representation of how the same systems, either loaded or unloaded, affect multiple different cell types (all arranged as 2D conformations—monolayers) in a different manner, in terms of concentration-dependent cytotoxicity.

**Figure 8:**
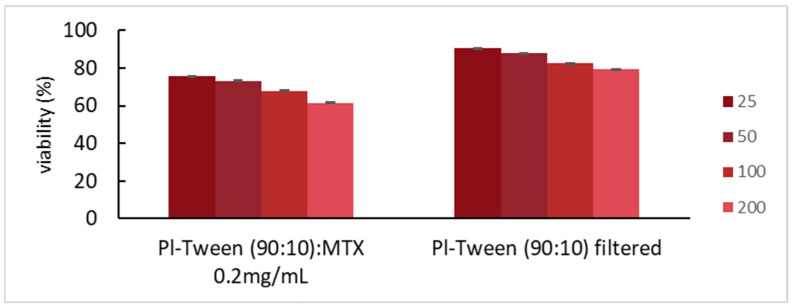
Cell culture viability vs. different concentrations of Pluronic 188–Tween 80 filtered nanosystems, loaded with the antitumor compound, methotrexate, and unloaded. The results shown are for HCT-116 3D spheroidal culture.

Multicellular tumor spheroids (mimicking the chaotic proliferation pattern of cell clusters) or three-dimensional cell culture systems are employed, amongst others, in order to obtain more accurate information on preclinical drug cytotoxicity. Additionally, 3D cultures are often used to study targeted therapies, interactions between specific cell populations, and for modeling various cellular mechanisms that are not yet fully understood. As such, spheroids have been shown to mimic to a greater extent the behavior of an in vivo solid tumor microenvironment when compared to 2D monolayer conformations (Figure 8).

As can be seen in Figure 7, the unloaded nano-carrier exhibits little to no cytotoxicity in concentrations below 100 μg/mL, while exhibiting the smallest slope in terms of viability/concentration dependence. Even at the lowest concentration, the unloaded carriers exhibit viability values below 95%, which might exhibit the cytostatic activity that often occurs after incubation with Poloxamer block copolymers, which are more easily internalized when filtered [28,29]. In the lowest concentration of nanosystem incubation (25 μg/mL), in all instances, the cellular viability after exposure to the loaded systems is lower than that of free MTX, indicating that perhaps at these quantities the nanosystem can act as a potential chemosensitizer for HCT-116 and SK-BR3 and HEK-293 cell lines, even though the latter is of a non-cancerous nature. This may be attributed to the poor bioavailability of MTX molecules, which, at low concentrations, the nanosystem enables to enter the cells more easily, rapidly causing the occurrence of a more toxic effect. In higher concentrations during incubation in such small-fixed volumes, it is possible that this effect is partially masked by the large MTX concentrations, which, even when partially internalized, can result in a highly toxic microenvironment [30].

While testing the nanosystem in multiple cellular models (both cancerous and normal), the presence of a circulatory system at the whole-organism level is vital in order to assess the nanoformulation’s indirect targeting capabilities through the EPR effect. Thus, the presented results between different cell lines cannot be correlated or indicative of the system’s effect in vivo. Through the statistical analysis of the experimental data (ANOVA comparing cellular type, nanosystem concentration, and nanosystem type), it is evident that while the concentration/viability correlation is of high statistical significance (*p* value < 0.0079 in all cases), the cellular type or viability/nanosystem type is of no significant consequence (*p* values = 0.4), thus providing further evidence that without the use of a living organism, the cellular selectivity of the nanosystem cannot be assessed.

It is important to stress that the concentration of MTX to which the cells were exposed, was in direct correlation with the dosages administered in today’s clinical practice (often ranging from 8 to 170 mg/m^2^ weekly, with more acute cases such as non-Hodgkin’s lymphoma reaching doses up to 8 g/m^2^). Since the surface area of a single well in a typical 96-well plate is 0.32 cm^2^, the equivalent in vivo doses were calculated based on the following equation and are presented in Table 2.

MTX concentration per well (mg) = 3.2–5 xequivalent clinical dose (mg/m^2^),

Given that the concentration of MTX successfully encapsulated in each nanosystem is roughly 1.7–1.8 mg per 10 mg of Pluronic 188 (based on the %EE as previously discussed §3.4).

While this approach acts as a crude estimation of dosage conversion between in vitro and in vivo experiment, it is important to note that there are multiple other parameters such as drug/organ interactions, API properties, ADME profile, etc., that need to be accounted for when proceeding with the further testing of novel pharmaceutical compounds [31].

### 2.7. Cellular Death Mechanism Assessment

**Figure 9:**
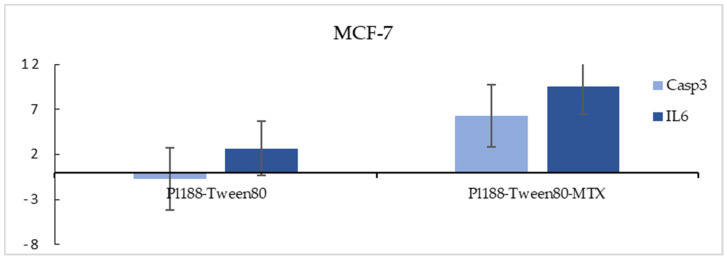
Fold change in mRNA levels in Casp3 and IL6 in a 2D culture of MCF-7 breast cancer adherent cell line for Pluronic 188–Tween 80 hybrid nanosystems, both loaded (right) and unloaded (left).

In order to examine the mechanism by which the nano-carriers achieve cellular death, several apoptosis-related genes were assessed in terms of their activity (possible upregulation or downregulation), after incubation with the nanocarriers for a period of 24 h at a fixed concentration of 200 μg/mL (represents the last point of the concentration/viability interval, equal roughly to a 100–120 mg/m^2^ encapsulated in vivo bioequivalent dose of MTX, assuming that all the nanosystems administered were internalized via endocytosis). Real-time quantitative PCR was performed in order to evaluate the mRNA levels of the expression of apoptosis-related genes, casp-3 and IL-6, in the MCF-7 breast cancer adherent cell line. Interestingly, an upregulation of IL-6 was achieved for both loaded and unloaded nanosystems, with the maximum change appearing in the loaded carriers, while Casp3 levels were slightly downregulated when cells were exposed to the unloaded carriers and upregulated when MTX was introduced to the system. p53 expression was not calculated since, as already mentioned, 4T1 cells are p53 null (Figure 9). While exhibiting a statistically significant effect on the regulation of either cancer-promoting or -inhibiting molecular pathways through proliferation or apoptotic gene regulation, it is important to note that, in most cases, the expression of such genes is only a part of a theoretical therapeutic approach, since enzymes that were originally thought to have a purely inhibitory response have now been linked to a lower tumor–drug sensitivity [25].

### 2.8. Monitoring Developmental Stages, Egg Laying, and Locomotion in Wild-Type C. elegans to Assess Toxicity

Wild-type N2 strains were used in order to examine the nanotoxicity effect of Pluronic 188–Tween 80 nanosystems, both loaded and unloaded, constituting an ideal animal model as an indicator of the adverse effects of multiple chemicals in mammalian species [32,33,34]. Two different controls were included in each assay—with the first (1) being nematodes with OP50 only and the second (2) being nematodes with Pluronic 188–Tween 80 unloaded nanosystems—since the empty nanocarrier comprises bioavailable, low-toxicity polymeric components, which showed low toxicity in the MTS assays. Control (1) was utilized as a baseline for all assays that followed.

**Figure 10:**
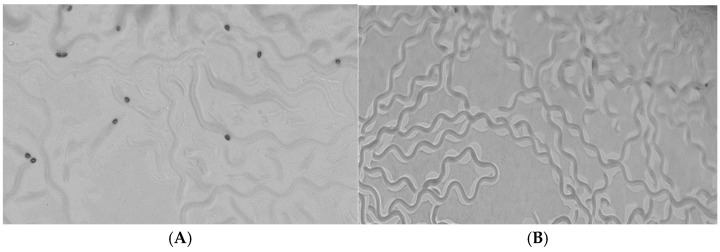
Representative plate images of (**A**) control (1) plate and (**B**) egg laying when nematodes where exposed to Pluronic 188–Tween 80–MTX nanosystems at a concentration of 150 μg/mL.

In these results, we accounted for the fact that a quantity of the colloidal nanoparticle suspension would diffuse through the agar and, as such, the concentrations tested were 5 μg/mL, 50 μg/mL (indicating no statistically significant change between the control groups and the groups exposed to the nanosystem in terms of nematode growth and development), and 150 μg/mL (Figure 10).

When exposed to the final concentration of 150 μg/mL, the results in terms of progenies were the following: control sample (1)—56 eggs, Pluronic 188–Tween 80; (2)—59 eggs, Pluronic 188–Tween 80–MTX; (3)—40 eggs, free MTX; and (4)—52 eggs. After roughly a 24 h period, L1 larvae or embryonic lethality, as well as the development of the above-mentioned eggs (48 h mark), were examined, and the following results were obtained: control sample (1)—32 larvae, Pluronic 188–Tween 80; (2)—28 larvae, Pluronic 188–Tween 80–MTX; (3)—21 larvae, free MTX sample; and (4) with 24 larvae (Table 3). These nematodes were evaluated in terms of possible growth delay, exhibiting abnormalities from the normal developmental cycle that *C. elegans* typically exhibits, indicating the toxicity effect of test groups (3) and (4).

**Figure 11:**
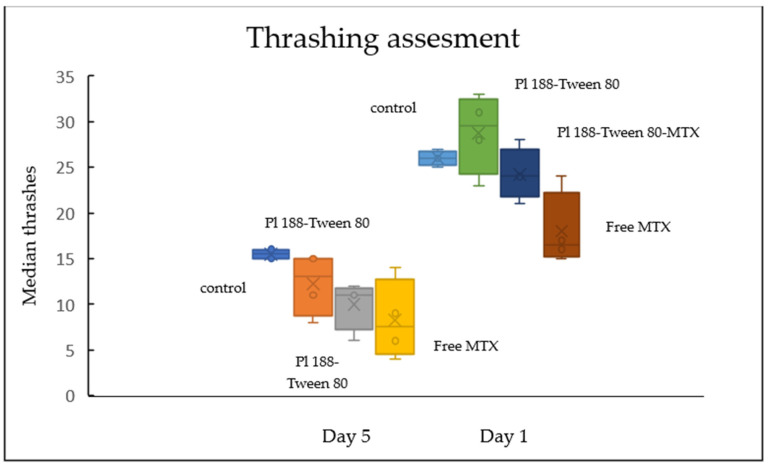
Toxicity assessment in 1-day and 5-day adult wild-type nematodes.

Lastly, we assessed the thrashing movement of both day 1 and day 5 adult worms (Figure 11), with the control (1) group exhibiting the least amount of diversity. These results indicate that the nematodes exposed to the methotrexate-loaded nanosystems exhibit statistically non-significant differences from the group exposed to free MTX. While nano-assemblies of block copolymers of the poloxamer family are able to act as chemosensitizers for multiple therapeutic compounds, including MTX, the release rate of the physically encapsulated active molecule is delayed and can be detected after the initial 4–5 h (Figure S6). These results are in accordance with the previous statement, since the experimental protocol in various instances indicated exposure times lower than 5 h. In addition, our experiments evaluated the release rate of the chemotherapeutic compound at an elevated temperature simulating human physiological conditions (37 °C), with indications that when the temperature drops below the lower critical solution temperature (LCST) of poloxamer block copolymers (20 °C-*C. elegans* culture), the release is further halted (the system’s metastable phase of Pluronic 188–Tween 80 (9:1) hybrid nanosystems, approximately between 30 and 35 °C, was previously determined through mDSC measurements) [16,35,36].

## 3. Materials and Methods

### 3.1. Materials

Poloxamer 188 (PLX 188, in the form of white microbeads), Tween 80^®^ (Polysorbate 80), Span 60^®^ (Sorbitan stearate), Span 40^®^ (Sorbitan Monopalmitate), and Methotrexate (in the form of yellow powder) were purchased from Sigma-Aldrich (Merck Group). Analytical-grade chloroform (CHCl_3_) as the organic solvent was purchased from Fisher Chemical TM. Bottled water (used for the injection) was purchased from DEMO AΒΕΕ, Athens. Phosphate-buffered saline tablets (PBS, 98%) and fetal bovine serum (FBS) were received from Sigma-Alrdrich, Athens, Greece. HEK293 non-cancerous cell line ATCC 293 CRL-1573™, SK-BR3 invasive epithelial cancer cell line, MCF-7 human breast cancer cell line, and HCT116 human colon cancer cell line were provided by ATCC (American Type Culture Collection) 10801 University Blvd, Manassas, USA [15].

### 3.2. Methods

#### 3.2.1. Preparation of Poloxamer 188/Surfactant and Poloxamer 188/Surfactant/MTX Colloidal Dispersions

Four distinct hybrid nanosystems—Pluronic 188–Tween 80 (90:10); Pluronic 188-Span 40 (90:10); and Pluronic 188-Span 60 (50:50)—were formulated using the thin-film hydration technique as described previously [16]. The final nanosystem Pluronic 188–Tween 80–MTX (9:1:0.2) was produced using the same technique. Appropriate volumes of the stock solutions in organic solvent (CHCl_3_) were mixed and placed in spherical flasks, which were inserted in the rotary evaporator (Hei-VAP series CORE-heidolph^®^) for a period of 4 h at a higher temperature between 50 ° and 60 °C and at reduced pressure until the the dehydrated thin film matrix formed on the walls of the flask after the evaporation of the organic solvent. The film was left to rest for 24 h at a fixed temperature in order to dry the remaining traces of the organic solvent. Afterwards, the hydration of the film took place using water for injection (DEMO^®^) at a final Pluronic 188 concentration of 10 mg/mL. Each sample was placed inside a sonication bath, following a specific protocol (3 min sonication, 2 min rest period, 2 min additional sonication), with the temperature not exceeding 40 °C. Hydrophilic Millipore^®^ syringe filters were used for filtering water before hydration. The final solution was transferred in sterilized glass vials and stirred at 500–600 rpm for a period of 45 min in order to achieve a final micellar dispersion (transparent yellow color). Finally, the obtained solution was filtered using a 0.22 μm hydrophilic filter membrane, thus removing the unincorporated MTX aggregates, with the remaining MTX being physically entrapped in the hydrophobic core of the amphiphilic nanosystem [17,36].

#### 3.2.2. FBS and FBS/PBS Interactions with Poloxamer 188/Surfactant Nanosystems

The colloidal dispersions of Pluronic 188/Surfactant were prepared using filtered PBS–nanoparticulate mixtures stored at a constant temperature (4 °C). Then, 50 μL of each nanoparticulate sample was diluted in 1.95 mL of FBS/PBS solution (1/9 *v*/*v* ratio) reaching a final volume of 2 mL. The mixed solutions were left for 1 h in stable conditions before measurements were conducted through light scattering techniques. Afterwards, the nanosystem exhibiting the most promise was evaluated in terms of physiological stability in plasma conditions by taking measurements at multiple time intervals (every 10 min) after FBS dilution [37]. Fetal bovine serum was chosen as biological media due to the presence of fetal bovine albumin (BSA), which is highly comparable with human serum albumin (HSA), a protein that is abundant in the blood compartment under normal conditions.

#### 3.2.3. Light Scattering Methods

The physicochemical characteristics of the formulated nanosystems (colloidal dispersion in aqueous media), such as size, size distribution, and scattered intensity, were evaluated using dynamic light scattering (DLS). The dilution protocol followed for the insertion of each colloidal dispersion in the sample cell was 50 μL of sample in 2 mL of filtered water for injection (0.45 μm hydrophilic pores). The hydrodynamic radius (Rh), size distribution (polydispersity index, PDI), and scattered intensity (I) were evaluated at a fixed temperature of 25 °C and at a scattering angle of 90° degrees. All experiments were replicated thrice. All measurements were performed with a wide-angle light scattering photometer by ALV GmbH, CGS-3, which is able to perform dynamic and static light scattering experiments simultaneously (National Hellenic Research foundation of Athens, Institute of theoretical and physical chemistry, Athens, Greece). This setup comprised a He-Ne 22 mW laser source, a compact goniometer system, an Avalanche photodiode detector interfaced with an ALV/LSE-5003 electronics unit, and an ALV-5000/EPP multi-tau digital photon correlator.

#### 3.2.4. Attenuated Total Reflectance—Fourier-Transform Infrared Spectroscopy (ATR-FTIR)

FTIR spectra of dry solid MTX sample, along with Pluronic 188–Tween 80–MTX samples, were attained as an average of 64 scans per spectrum in the range from 5000 to 500 cm^−1^ at a working resolution of 4 cm^−1^, in order to chemically verify the structure and MTX encapsulation of each sample. A Bruker (Billerica, MA, USA) Equinox 55 Fourier-transform spectrometer was used for the analysis (National Hellenic Research foundation of Athens, Institute of theoretical and physical chemistry, Athens, Greece), along with a single-bounce ATR diamond accessory (Dura-Samp1IR II, SensIR Technologies, Danbury, CT, USA). All measurements were conducted at a fixed temperature of 25 °C. The resulting ATR-FTIR spectra of the formulated nanosystems exhibited new peaks specific to methotrexate.

#### 3.2.5. Ultraviolet–Visible (UV–Vis) Spectroscopy

The determination of drug encapsulation efficiency (%EE) and drug loading (%DL) was evaluated via UV–Vis spectroscopy(National Hellenic Research foundation of Athens, Institute of theoretical and physical chemistry, Athens, Greece). After dilution of each sample in acetonitrile, the absorbance was recorded at 303 nm, with the EE and DL being calculated for the range from 0.025 mg/mL to 0.8 mg/mL after the formation of the standard curve for MTX, diluted using the same media.

#### 3.2.6. Dialysis Bag—Drug Release Studies

The amount of the encapsulated MTX released from the nanosystem was evaluated in different time intervals, under different temperature and pH conditions, with distinct release mediums (PBS and 1/9 FBS/PBS). A total of 1 mL of Pluronic 188–Tween 80 colloidal dispersion (containing 0.2 mg/mL MTX) was introduced in a sealed dialysis bag (MWCO = 3500 Da), immersed in 9 mL of release medium. The incubation media were all withdrawn and replaced with a pre-warmed fresh medium at multiple time points, thus being able to maintain optimal sink conditions [38]. The medium was subjected to mechanical steering via a Teflon-coated magnet (100 ± 10 rpm), from 0 min until the end of the experiment.

#### 3.2.7. MTS Cytotoxicity Assay

##### 2D Monolayer In Vitro Nanotoxicity

HEK293, SK-BR3, MCF-7, and HCT116 cells—ATCC (American Type Culture Collection) 10801 University Blvd, Manassas, USA—were cultured in suitable media comprising DMEM High Glucose (BioSera, Shanghai, China) mixed with 10% FBS (PAN Biotech, Aidenback, Germany), along with 100 U/mL penicillin and 100 g/mL streptomycin. Cells were incubated at a fixed temperature of 37 °C and a 5% containing CO_2_ atmosphere, using a steri-cycle CO_2_ incubator (HEPA Class 100, Thermo Electron Corporation^®^). Every 48 h, the medium was replaced, and the cells were passaged on a weekly basis using the trypsin/EDTA method (≤30 number of passages). At ≥85% confluency, cells were transferred to a 96-well plate, and 5000 cells/well were seeded. All procedures took place in a sterile environment. Cultures were exposed to multiple nanoformulation concentrations for a period of 24 h under the same conditions. Incubation took place using sterile microplates under a sterile hood. All experiments were duplicated (*n* = 2), while each analysis took place less than a week from nanocarrier synthesis [39].

##### In Vitro Nanotoxicity Using 3D Spheroid Culture (Multicellular Tumor Spheroids)

HCT116 human colon cancer cells were cultivated using a polymeric agarose scaffold into a final 3D structure. Next, 1% (*w*/*v*) agarose (Low-gelling-temperature Agarose, Nippon Genetics Europe GmbH, Düren, Germany) gel was formulated using 1 X TAE as a buffer, with a final density of 104 cells/well immobilized. A 1.5 mL aliquot of the mixture was loaded into a 96-well plate and kept at room temperature for 30 min to allow the gel to solidify. Afterwards the cells were cultured using DMEM High Glucose medium containing 10% FBS and 1% penicillin and streptomycin. Following, the 6-well plates were centrifuged for a period of 1 min at 10.000 rpm. Fluorescence was measured after a 24 h incubation period of the nanosystems, using a multi-detection reader (BioTek 800 TS Elx800), provided by the laboratory of biology, Department of medicine, Athens, Greece.

#### 3.2.8. Real-Time PCR Assay for Caspase 3 (Casp3) and Interleukin 6 (IL6) Expression Assessment

RNA extraction (for Pluronic 188–Tween 80–MTX concentration of 200 μg/mL) was executed using TRIzol reagent (Thermo Fisher Scientific, Chalandri, Greece), with respect to the manufacturer’s instructions. PrimeScript First Strand cDNA Synthesis Kit (TAKARA) was used for reverse transcription with the reaction’s conditions being 37 °C for 30 min followed by 85 °C for 5 s. Thermal Cycler (Kyratec Super Cycler, laboratory of biology, Department of medicine, Athens, Greece) was used for the performance of the reaction. MCF-7 human breast cancer cell line was cultivated and exposed for 24 h to Pluronic 188–Tween 80–MTX and to empty Pluronic 188–Tween 80 filtered carriers, after which the cells were lysed and the RNA was extracted. The expressions of IL6 and Casp3 apoptotic genes were evaluated and compared with those of the untreated cells. The expression of p53 was not evaluated.

Quantitative real-time RT-PCR was conducted on an ABI Prism 7000 apparatus (Applied Biosystems, Foster City, CA, USA). All extracted samples were mixed with appropriate primer sets along with PCR master mix (KAPA SYBR FAST qPCR Kit). Gene expression was normalized by subtracting the Ct value of the GAPDH RNA internal control from that of the GOI (gene of interest) (ΔCt = −|CtGOI-CtGAPDH|). The relative expression of GOI in cancer cells compared to non-cancer cells was the 2ΔΔCt model, where ΔΔCt = ΔCtGOI-ΔCtGAPDH [40].

#### 3.2.9. In Vivo Toxicity Studies—*Caenorhabditis elegans* Culture

The N2 strain of *C. elegans* wild-type Bristol isolate was utilized in order to evaluate the comparative nanotoxicity of the formulated nanosystems (Pluronic 188–Tween 80 unloaded and loaded carriers at a concentration of 150 mg/L), along with free MTX. Nematodes were maintained at 20 °C (AQUA^®^ LYTIC incubator) on 6 cm Petri plates containing Nematode Growth Medium (NGM), spotted with Escherichia coli OP50 bacteria as a food source (stored at a fixed temperature of 4 °C). Prior to supplementing with nanosystems, the bacterial lawn was UV-irradiated for 15 min to prevent the potential metabolism of the nanoparticles by the bacteria. Nematode assessment and handling was completed with the use of a dissecting stereomicroscope (Nikon, model: SMZ645).

##### Assessment of Egg Laying, Embryonic Lethality and Developmental Stages

Synchronized young adult nematodes (synchronized by picking L4 larvae from the N2 strain) were exposed to the nanoformulations for 3 h, after which all nematodes were transferred to different plates containing equal concentrations of the nanoformulations. In each plate, previously containing 10 N2 early-adult worms, the number of eggs were counted, and the respective results were expressed as a mean number of eggs laid per animal. The number of hatched nematodes were counted again 24 h after the initial incubation in order to assess the number of eggs that did not produce any viable nematodes.

##### Locomotion Assay

Head thrashing in *Caenorhabditis elegans* is defined as the bending of the animal’s head until it reaches at least a quarter of its body length. For observation, each nematode was placed in a 10 μL drop of M9 solution on a siliconized microscope slide and observed through a dissecting stereomicroscope. A 30 s acclimation period was provided for each animal before locomotion recording began. The following 30 s of movement was manually recorded using a cell counter [41,42,43,44].

#### 3.2.10. Future Steps and Promises for Clinical Practice

In today’s clinical practice, the primary tools of almost every treatment plan and therapeutic option against solid tumor proliferation are chemotherapy, radiotherapy, and medical surgery, approaches that in many cases and cancer phenotypes yield mostly unsatisfactory results, while some have the disadvantage of being more invasive than what would optimally be preferable. The successful implementation of a novel nanoparticulate system, such as the one described in this manuscript (complementary with already existing therapeutic protocols), can offer significant benefits for the clinical outcomes of many cases if it yields satisfactory clinical results (a positive yield of therapeutic effect vs. side effects). The developed hybrid nanosystem would have the ability to deliver a chemotherapeutic drastic substance directly to the target-cells via the enhanced retain and permeability effect (EPR), sparing the rest of the organism and healthy cells from the cytotoxic and genotoxic activity of the chemotherapeutic substance. At the same time, since a higher relative concentration of the API will be delivered to the cancerous cells, a lower overall concentration will need to be administered, further minimizing the side-effect profile. In addition, the prolonged release of the encapsulated drug helps to avoid drastic changes in drug blood plasma concentration (peaks and valleys), thus prolonging the re-administration time between two therapy cycles. Lastly, the developed nanosystem may be further functionalized in order to incorporate hydrophilic compounds in its outer periphery (targeting moieties, monoclonal antibodies, PEG molecules, and additional therapeutic–antioxidant compounds), since it is a multi-compartmentalized system.

Lastly, it is important to note that the current study is not without certain limitations, including a more in-depth analysis of the possible gene regulation of apoptotic-related pathways, the expression of which may be affected by the final loaded nanoformulation. Also, while *C. elegans* represents a perfectly suitable model to assess the nanotoxicity characteristics of the current hybrid system, it does not offer the possibility to draw further in-depth data that studies in higher-order animals would provide (ADME profile, tumor suppression, immune system activation, blood plasma half-life, etc.).

## 4. Conclusions

A novel hybrid nanodelivery system was successfully designed and developed that was able to encapsulate the model drug, methotrexate, with a high loading capacity. Stable, prolonged release was observed, exhibiting the system’s ability to deliver BCS class II and IV molecules to multiple human cell lines. The nanosystem, comprising the triblock copolymer Pluronic 188 and the surfactant polysorbate 20 (Tween 20) at a *w*/*w* ratio of 9:1, showed physicochemical characteristics (size, PDI, HLB characteristics, micropolarity, microviscosity, etc.) that remained stable after prolonged incubation times with FBS/PBS media, an important trait for any system with the ability to avoid immune system recognition and circulate for prolonged periods of time inside the human body (i.e., absence of non-specific protein/nanoparticle interactions). Multiple light scattering techniques validated the high stability and loading capacity of the final formulation, as well as the advantage of a mean hydrodynamic particle diameter less than 200 nm with a low polydispersity index. The development of an amphiphilic system with a relatively high HLB ratio and sufficiently small size, while at the same time maintaining such high encapsulation efficiency, is an important feat, since it lowers the possibility of agglomeration and thus provides a system with a better risk profile. The release of the encapsulated MTX followed a controlled/delayed pattern, an attribute that can allow for a more stable blood plasma level maintenance, avoiding high peaks followed rapidly by low valleys (thus avoiding the occurrence of dose-related side effects). In vitro experimentation in multiple cell types and culture conformations (2D and 3D spheroid cultures) indicated a dose-dependent toxicity, while the empty nanocarriers resulted in the greatest viability values, as was anticipated. In vivo experiments on the model organism *C. elegans* N2 wild-type strain stressed the toxic effects of free methotrexate exposure at higher concentrations, which are exhibited by the loaded carrier after the 5 h window at physiological conditions (37 °C), when drug release starts to occur. This is the first time that such a system has been holistically examined, from the earliest formulation stages (physicochemical characterization) up to in vivo experimentation, and presented in one complete manuscript, exhibiting how multiple parameters may affect the final nano-drug product. The final novel nanosystem might act as a possible chemosensitizer, while obtaining physicochemical characteristics that enable its possible use for the encapsulation of multiple chemotherapeutic APIs that belong to the class II and BCS IV BCS. Characteristics such as a prolonged release rate, can result in a preferable clinical effect, augmenting the time intervals between two consequent administrations, ameliorating the phycological well-being of the patient through the minimization of the hospitalization period. Lastly, the current investigation can be utilized as a tool to further study poloxamer/surfactant interactions for the formulation of nano-drug carriers of a similar nature through the thin-film hydration technique, exhibiting spatial compartmentalization and the ability to encapsulate high doses of both hydrophilic and lipophilic APIs. The hybrid nanosystem at hand can be further evaluated pre-clinically, in order to assess its possible incorporation into today’s therapeutic protocols, ameliorating the effects of more conventional treatments.

## Figures and Tables

**Table 1:** Physicochemical characteristics of the loaded Pluronic 188–Tween 80 hybrid nanosystems (filtered).

System	MTX (mg/mL)	R_h_ (nm) ^a^	I (KCps) ^b^	PDI ^c^
Poloxamer 188–Tween 80 (90:10)	-	65	101	0.323
Poloxamer 188–Tween 80 (90:10)	0.1	66	151	0.280
Poloxamer 188–Tween 80 (90:10)	0.2	64	151	0.219
Poloxamer 188–Tween 80 (90:10)	0.3	88	30	0.546

^a^ R_h_(nm): Hydrodynamic diameter; ^b^ I(KCps): Scattering intensity; ^c^ PDI: polydispersity index.

**Table 2:** In vitro concentration calculation.

In Vitro MTX Concentration (μg/mL)	In Vivo Equivalent Dose (mg/m^2^)
0.5	16
1	32
2	64
4	128

**Table 3:** Embryonic lethality assessment after exposure to various nanoformulations and free MTX.

	Control (1)	Pluronic 188–Tween 80 (2)	Pluronic 188–Tween 80-MTC (3)	Free MTX
Eggs	56	59	40	52
Larvae	32	28	21	24
Embryonic viability (%)	57.2	47.5	52.5	46.2

## Data Availability

The original contributions presented in the study are included in the article/Supplementary Materials, further inquiries can be directed to the corresponding author.

## Supplementary Materials

The following supporting information can be downloaded at: https://www.mdpi.com/article/10.3390/ijms252111520/s1.
