# Supplementary material for: Chemotherapeutic Drug Delivery Nanoplatform Development: From Physicochemical to Preclinical Evaluation
Source: Int J Mol Sci. 2024 Oct 26;25(21):11520. doi: 10.3390/ijms252111520 (PMC11546407; doi:10.3390/ijms252111520)
Supplement: Supplementary file 1 [file ijms-25-11520-s001.zip › ijms-3254603-supplementary.pdf]

Supplementary Material

Article

Chemotherapeutic Drug Delivery Nanoplatform Development: From Physicochemical to Preclinical Evaluation

Orestis Kontogiannis [1,2], Dimitrios Selianitis [2], Konstantinos Palikaras [3], Natassa Pippa [4], Stergios Pispas [2], Efstathios Efstathopoulos [5] and Maria Gazouli [1,5,*]

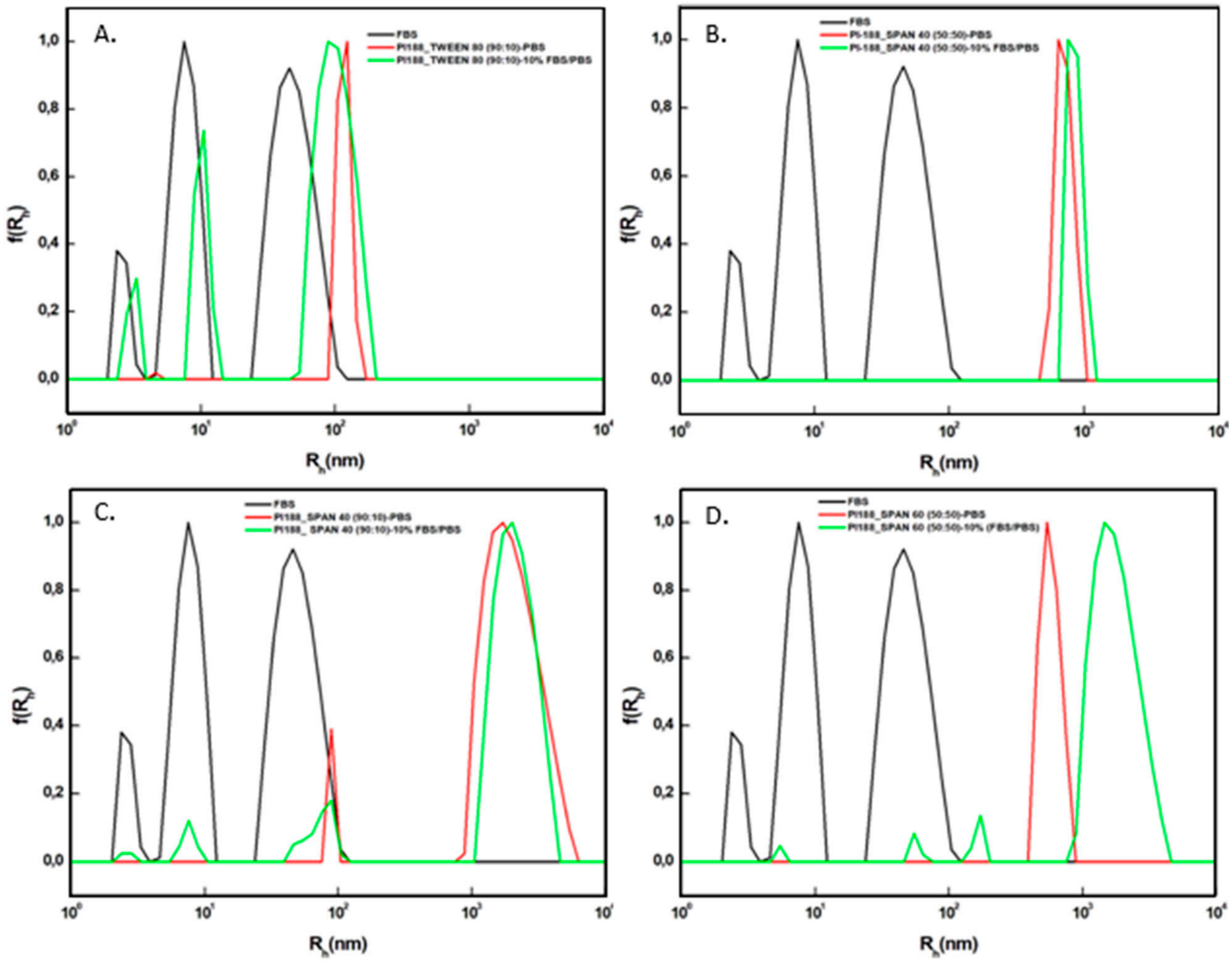

Figure S1. Size distribution graphs of Pluronic 188–surfactant nanosystems (unfiltered) under physiological conditions. Each depicted curve is the result of the average of three measurements. $C_{Pluronic} = 1 \times 10^{-3}$ g/mL: A, the stability of Pluronic-Tween 80 (90:10) nanosystem; B, Pluronic–Span 40 (50:50) nanosystem; C, Pluronic–Span 40 (90:10) nanosystem; and D, Pluronic–Span 60 (50:50) nanosystem.

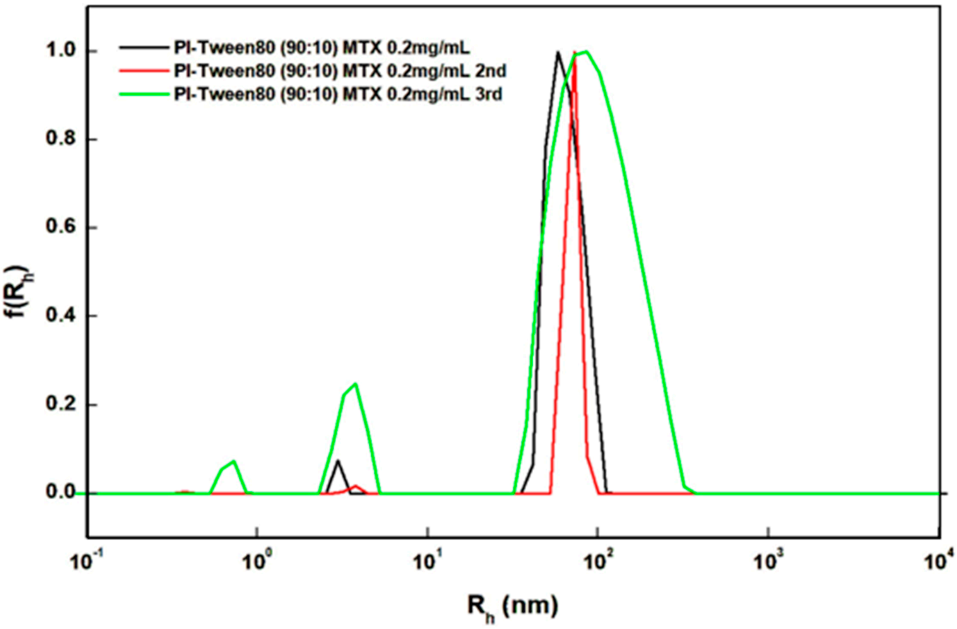

Figure S2. Comparative size distribution graphs of Pluronic 188–Tween 80–MTX (0.2mg/mL) using the thin-film hydration technique. As depicted, the formulation process was replicated thrice, producing highly comparable results.

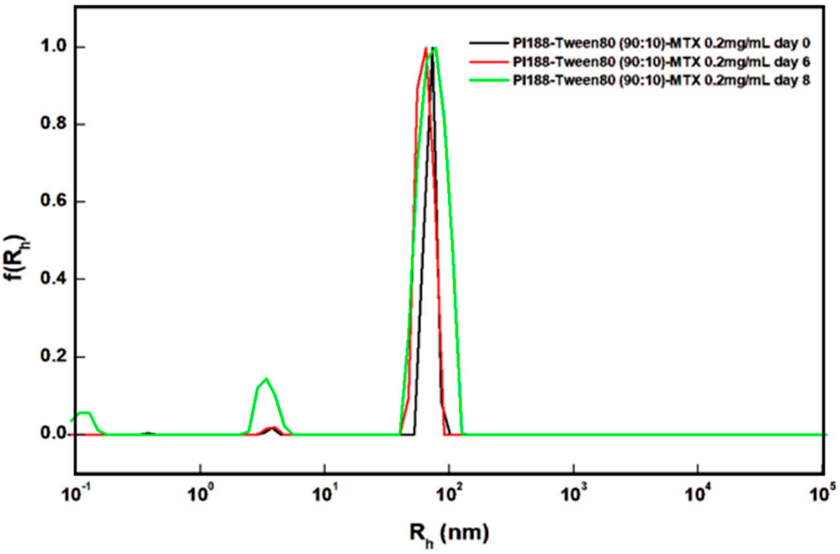

Figure S3. Stability assessment of the formulated Pluronic 188–Tween 80 (90:10) nanosystems with 0.2mg/mL MTX encapsulated over time.

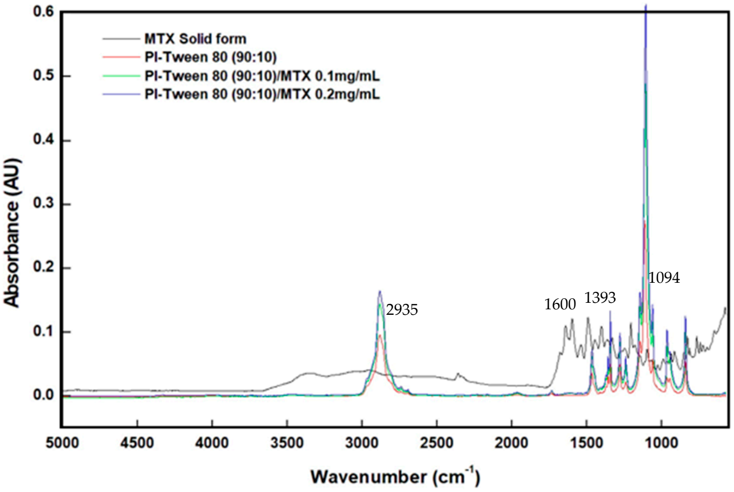

Figure S4. FTIR spectra of free MTX, Pluronic 188–Tween 80, and Pluronic 188–Tween 80–MTX loaded nanosystems.

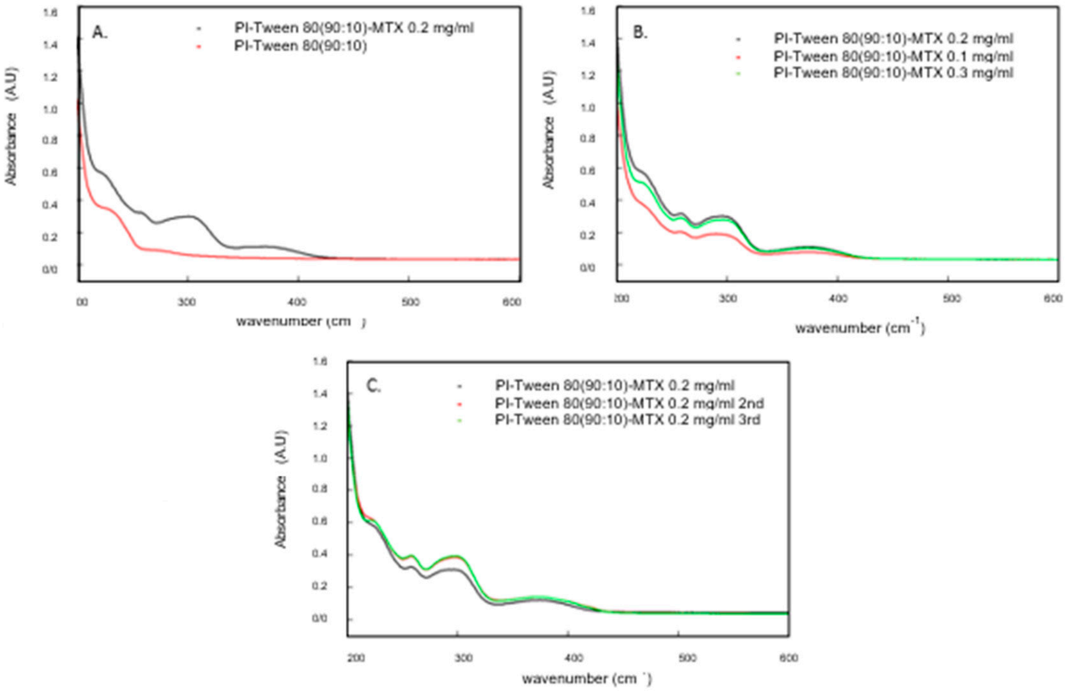

Figure S5. Encapsulation efficiency assessment through UV–Vis spectroscopy: A, comparative plots of the hybrid nanosystem unloaded and loaded with MTX 0.2mg/mL; B, comparative plots of different MTX concentrations; and C, reproducibility assessment (see figure insets for details).

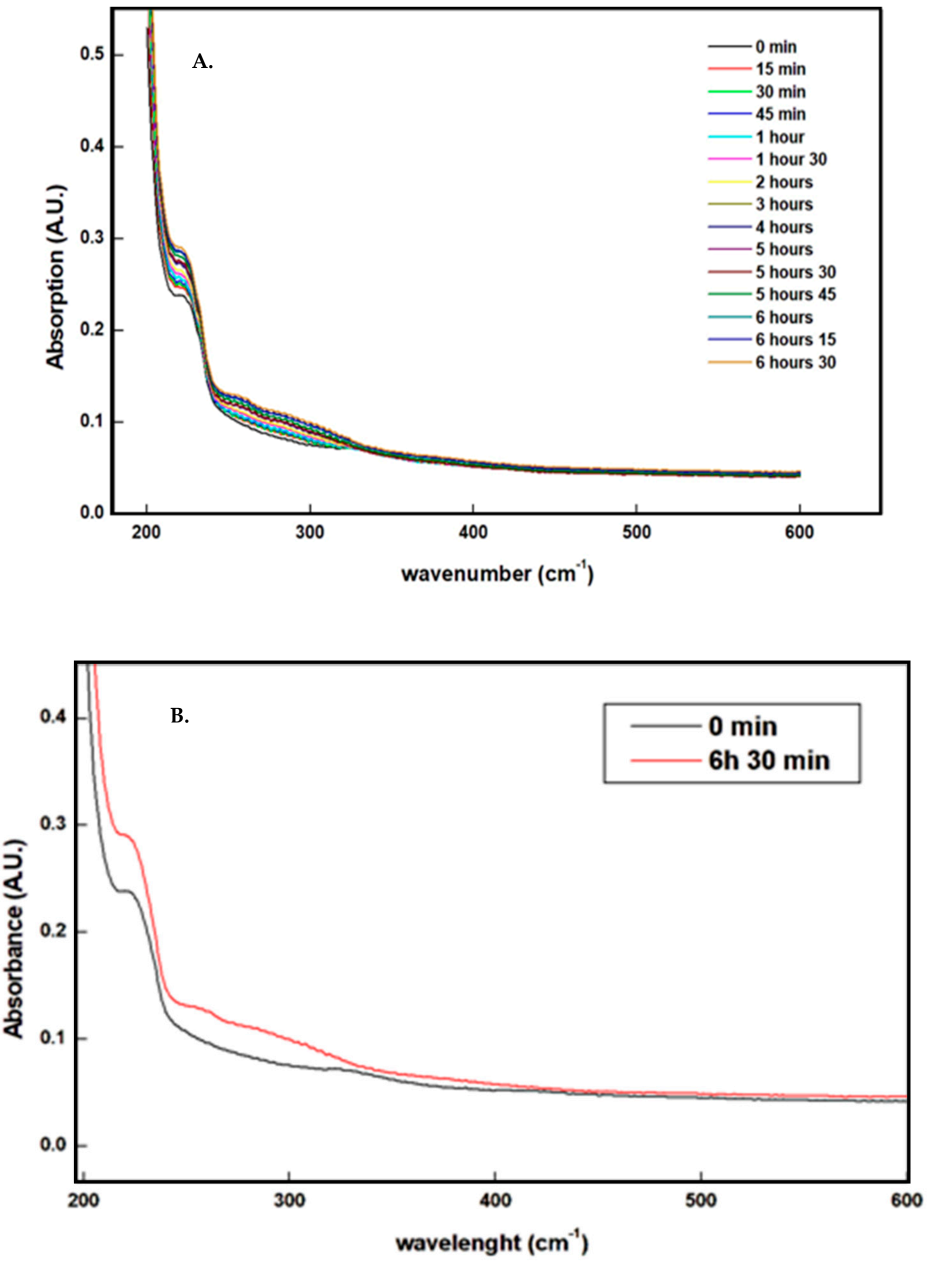

Figure S6. In vitro drug release study of MTX (303nm) in PBS media at 37ºC, where A represents the whole progression over time, and B exhibits the total difference between the initial (T=0) and final (T= 6h 30min) time point of the experiment.

Table S1. Encapsulation efficiency results (%EE) for 0.1 mg/mL, 0.2 mg/mL, and 0.3 mg/mL MTX.

MTX = 0.1mg/mL	MTX = 0.2mg/mL	MTX = 0.3mg/mL
y=1,462x + 0,084	y=1,462x + 0,084	y=1,462x + 0,084
0,195 = 1,462x + 0,084	0,305 = 1,462x + 0,084	0,283 = 1,463x + 0,084
1,462x = 0,115	1,462x = 0,221	1,462x = 0,2
x = 0,0759	x = 0,1511	x = 0,1363
%EE = 75.9%	%EE = 75.6%	%EE = 45.33%
